# Supplementary material for: Hypothalamic-Pituitary-Ovarian Axis Disorders Impacting Female Fertility
Source: Biomedicines. 2019 Jan 4;7(1):5. doi: 10.3390/biomedicines7010005 (PMC6466056; doi:10.3390/biomedicines7010005)
Supplement: Supplementary file 1 [file biomedicines-07-00005-s001.pdf]

	Disorders	Etiology	Work-up	Management
**WHO Classification**<br>Group I: Hypogonadotropic Hypogonadal Anovulation	**Idiopathic hypogonadotropic hypogonadism (IHH)**	***Normosomic***<br>Mutations of GNRHR, ANOS1, FGF8/FGFR1, TACR3, CCDC141, NELF[4]<br>***Kallmann***<br>Interrupted GnRH neuron migration from nasal placode; FGFR1, ANOS1 (KAL1), FGF8, FGF17, IL17RD, DUSP6, SPRY4, FLRT3, KLB, PROK2, PROKR2, HS6ST1, CHD7, WDR11, SEMA3A, SEMA3E, IGSF10, SMCHD1, CCDC141, FEZF1, HESX1	- Brain MRI<br>- FSH, LH	- Transdermal estradiol patch; gradually increase dose<br>- Conjugated equine estrogen with Cyclic medroxyprogesterone 10mg 12-14 days/month<br>- combined oral contraceptive pill<br>- Fertility treatment: recombinant FSH, human menopausal gonadotropin
	**Septo-Opto Dysplasia**	- Vascular insult in 6th-7th week of embryogenesis<br>- HESX1 mutation<br>- Associated risk factors:<br>- 1st trimester bleeding<br>- Illicit drug use<br>- Primiparity	- Brain MRI<br>- FSH, LH	- Neurodevelopmental monitoring<br>- Combined oral contraceptive pill<br>- Estradiol patch or oral conjugated equine estradiol with cyclic medroxyprogesterone
	**Panhypopituitarism**	- Pituitary tumors<br>- Traumatic brain injury<br>- Inflammatory changes ((Mycobacterium Tuberculosis, histoplasmosis, syphilis, viruses, protozoa, lymphocytic hypophysitis)<br>- Infiltrative disease (Histiocytosis, hemochromatosis, sarcoidosis)<br>- Pituitary apoplexy, Sheehan's syndrome<br>- Congenital (genetic mutation of HESX1, PROP1, and Pit-1)<br>- Empty Sella Syndrome	- Brain MRI<br>- FSH, LH<br>- TSH, ACTH	- Combined oral contraceptive pill<br>- Estradiol patch or oral conjugated equine estradiol with cyclic medroxyprogesterone<br>- Correct other endocrinopathies<br>- Fertility treatment: recombinant FSH, human or menopausal gonadotropin
	**Craniopharyngioma**	- Embryonic theory: arises from remnants of Rathke's pouch<br>- Metaplastic theory: metaplasia of squamous epithelial cell rests from stomodeum<br>- Mutations in CTNNB1 that encodes β-catenin	- Brain MRI<br>- FSH, LH	- Radiotherapy or subtotal resection<br>- Fertility treatment: recombinant FSH, human menopausal gonadotropin
	**Langerhans Histiocytosis X**	- Somatic mutations gain of function in BRAF gene in 50% of cases	- Brain MRI<br>- FSH, LH<br>- Prolactin, TSH, ACTH<br>- Basic metabolic profile	- Radiotherapy<br>- Correct endocrinopathy<br>- Fertility treatment: human menopausal gonadotropin (hMG)
**WHO Classification**<br>Group II: Irregularities in HPO Axis	**Polycystic Ovarian Syndrome**	- Multifactorial<br>- Candidate genes mutated: GLUT4, miR-93	- Total testosterone<br>- 17-hydroxyprogesterone<br>- Dehydroepiandrostene-dione sulfate<br>- 2-hour glucose tolerance test<br>- Lipid panel<br>- Transvaginal ultrasonography	- Combined oral contraceptive pill<br>- Fertility treatment: First line- letrozole Second line- clomiphene citrate
	**Hyperprolactinemia**	- Prolactinoma<br>- Primary hypothyroidism<br>- Autoimmune disorders<br>- Chronic renal failure<br>- Psychotropic medications	- Prolactin<br>- TSH<br>- MRI of the pituitary fossa	- Cabergoline<br>- Bromocriptine<br>- Surgical decompression if medical management fails
	**Thyroid dysfunction**	***Hyperthyroidism***<br>- Hashimoto's thyroiditis<br>- Lymphocytic thyroiditis<br>- Radiation therapy<br>- Surgery<br>- De Quervain<br>- Infiltrative sarcoidosis<br>- Hemochromatosis<br>- Drugs (Lithium, Amiodarone)<br>***Hypothyroidism***<br>-Graves disease<br>- Iatrogenic (drug induced, TKI, INFα)<br>- Inadequate Iodine<br>- Struma ovarii<br>- Infection	- TSH<br>- Total and free T4<br>- Consider total and free T3	- Levothyroxine if hypothyroid<br>- Methimazole or propylthiouracil if hyperthyroid
	**Endometriosis**	- Multifactorial<br>- miR-135a overexpression<br>- Copy number variants: 1p36, 7p22.1 and 22q12	- Diagnostic laparoscopy	- Surgical ablation of lesions<br>- Ovulation induction agents
**WHO Classification**<br>Group III: Hypergonadotropic Hypogonadal Anovulation	**Primary Ovarian Insufficiency**	- Genetic (Turner's, Fragile X)<br>- Acquired (autoimmune, environmental)<br>- Iatrogenic (chemotherapy agents, drugs, brain radiation)	- AMH<br>- Karyotype<br>- FMR1 pre-mutation	- Combined oral contraceptive pill<br>- Estradiol patch or oral conjugated equine estradiol with cyclic medroxyprogesterone<br>Fertility treatment:<br>- Oocyte or ovarian tissue* cryopreservation<br>- IVF with donor oocytes<br>- Stem cell therapy*<br>- AUGMENT*<br><br>*still considered experimental

Table S1. Summary of group I-III ovulation disorders including etiology, evaluation and management.
